# Association of tryptophan hydroxylase-2 polymorphisms with oppositional defiant disorder in a Chinese Han population

**DOI:** 10.1186/s12993-016-0113-0

**Published:** 2016-11-21

**Authors:** Chang-Hong Wang, Cong Liu, En-Zhao Cong, Gai-Ling Xu, Ting-Ting Lv, Ying-Li Zhang, Qiu-Fen Ning, Ji-Kang Wang, Hui-Yao Nie, Yan Li

**Affiliations:** 1 Department of Psychiatry, The Second Affiliated Hospital of Xinxiang Medical University (Psychiatric hospital of Henan province, China), Xinxiang, 453002 Henan China; 2 Department of Child and Adolescent, Public Health College, Zhengzhou University, 100 Kexue Road, Zhengzhou, 450001 Henan China

**Keywords:** Oppositional defiant disorder, Tryptophan hydroxylase-2 gene, Single nucleotide polymorphisms

## Abstract

**Background:**

Oppositional defiant disorder (ODD) is a behavioral disorder of school-age population. It is well known that 5-HT dysfunction is correlated with impulsivity, which is one of the common characteristics of ODD. The enzyme tryptophan hydroxylase-2 (TPH-2) synthesizes 5-HT in serotonergic neurons of the midbrain raphe. The purposes of this study were to investigate the potential association of *TPH*-*2* polymorphisms with susceptibility to ODD in a Han Chinese school population.

**Methods:**

Four polymorphisms (rs4570625, rs11178997, rs1386494 and rs7305115) of the TPH-2 gene were analyzed by using polymerase chain reaction and DNA microarray hybridization in a case–control study of 276 Han Chinese individuals (124 ODD and 152 controls).

**Results:**

In single marker analyses,there was a significant difference in the genotype (*χ* ^*2*^ = 4.163, *P* = 0.041) and allele frequency (*χ* ^*2*^ = 3.930, *P* = 0.047) of rs1386494 between ODD and control groups. Haplotype analyses revealed higher frequencies of haplotypes TA (rs4570625-rs11178997), TAG (rs4570625-rs11178997-rs1386494), TAA (rs4570625-rs11178997-rs7305115) and TAGA (rs4570625-rs11178997-rs1386494-rs7305115), but lower frequencies of haplotypes GA (rs4570625-rs11178997) and GAG (rs4570625-rs11178997-rs1386494) in ODD compared to control groups.

**Conclusions:**

These findings suggest the role of these *TPH*-*2* gene variants in susceptibility to ODD. Some haplotypes might be the risk factors for Chinese Han children with ODD, while others might be preventable factors.

## Background

Oppositional defiant disorder (ODD) is a behavioral disorder mainly characterized by resistance, disobedience, provocation or hostility to authority figures during growth and development in children and adolescents [1, 2]. Children and adolescents with ODD may have trouble controlling their temper, showing intense emotional reaction or impulsive actions in response to mild stimulation. Thus, ODD is considered as a disorder of emotional regulation [3]. Children suffering from ODD are at risk for numerous negative outcomes, such as delinquency, unemployment, depression, anxiety and other psychiatric problems [4]. However, the pathological mechanisms of ODD are still unclear.

Based on DSM-IV-TR, the prevalence rate for ODD is 2–16% [5]. Well established in previous research, this disorder exhibits moderate heritability, and is substantially stable over time, particularly through childhood [6], and genetic underpinning is an important factor which can influence children’s disruptive behavior, like ODD [7]. Familial clustering suggests an underlying genetic component, but hereditary connections are variable [1]. ODD has been consistently associated with attention-deficit/hyperactivity disorder (ADHD) [8, 9] and conduct disorder (CD) [10–13]. The estimated heritability of ADHD is approximately 0.76 [14] and 40–60% of ADHD were also diagnosed with ODD [15], suggesting that ODD might share common genetic mechanisms with ADHD [8]. However, the comorbidity of ODD may influence the clinical characteristics, progression and treatment response for ADHD cases [14].

Serotonin (5-HT) is a neurotransmitter involved in various bodily functions, such as aggression, attention, appetite and locomotion. The deficiency of the 5-HT functions is related to depression, anxiety, irregular appetite, aggression, increased pain sensation, and ADHD symptoms [16]. Especially, 5-HT dysfunction is correlated with impulsivity, which is one of the common characteristics of ADHD, ODD, personality disorder [17] and substance abuse [18, 19]. Early studies reported a clear association between low cerebrospinal fluid 5-HT and impulsive aggression [20]. The conversion of tryptophan to 5-hydroxytryptophan is the first and rate limiting step in 5-HT synthesis catalyzed by two subtypes of the enzyme tryptophan hydroxylase (TPH-1 and TPH-2); 5-HT is then formed by decarboxylation of 5-hydroxytryptophan. The studies revealed differential expression of classical TPH-1 synthesizing 5-HT in peripheral tissues, and TPH-2 synthesizing 5-HT mainly in serotonergic neurons of the midbrain raphe [21]. In mice brain stems, the expression of TPH-1 appears to be 150 times lower than TPH-2 [21], suggesting that TPH-2 may play a much more important role in serotonin synthesis in the brain than TPH-1. Thus, the studies have focused on the role of brain-specific TPH-2 in the pathophysiology of various psychiatric disorders, including ADHD [16].

The human TPH-2 gene spans less than 100 kb, consists of 11 exons and is located in the chromosome 12q21.1 region. Several studies have explored the association between TPH-2 gene polymorphisms and ADHD. For example, Sheehan et al. [22] firstly reported the association between *TPH2*-rs1843809 and ADHD through a family study. A subsequent study reported association between *TPH2*-rs4570625 or *TPH2*-rs11178997 and ADHD through a family study [23]. A more recent study showed that a significant correlation between the frequencies of the rs11179027 and rs1843809 of alleles of *TPH*-*2* and ADHD [16]. In addition, the TPH-2 gene polymorphism have been found to be associated with late-onset depression [24], PTSD [25, 26], suicide in patients with alcohol dependence [27, 28] and suicidal behavior [29], as well as with schizophrenia [30, 31] and panic in bipolar disorders [32] in the Chinese Han population.

In view of the possible shared common genes between ADHD and ODD, the important role of TPH-2 in 5-HT synthesis in brain and the possible association between TPH-2 gene polymorphisms and ADHD, as well as the associations of the TPH-2 gene polymorphism with behavioral and psychiatric disorders in previous studies, it would be of interest to examine the association between TPH-2 gene polymorphisms and ODD, which, to our best knowledge, has not been reported. Therefore, the main purpose of the current study was to examine whether the TPH-2 gene polymorphisms was associated with the susceptibility to ODD in a Chinese Han population.

## Methods

### Subjects

Using the random group sampling method, 2000 Chinese Han students in primary school in Nanyang, Henan Province, China were assessed with Conners Teachers Rating Scale between 2007 and 2009, all four grandparents and both parents of each child were known to be of Han Chinese origin. To confirm the diagnosis of ODD, one or both parents and teachers were interviewed by a chief physician and resident physician on the basis of DSM-IV diagnostic criteria. Inclusion criteria were: (a) aged 6–14 years; (b) had ODD symptoms at least 6 months; (c) intelligence quotient (IQ) ≥70 based on Raven’s Progressive Matrices; (d) no physical diseases, mental retardation, low body mass index (BMI, <18.5 kg/m^2^) or other mental illnesses, or ADHD or CD symptoms. A total of 125 children were confirmed with ODD diagnosis. Among them, 124 subjects were enrolled in the study, including 70 boys (56.5%) and 54 girls (43.5%) with an average age of 10.4 ± 1.9 years.

Data also consisted of 152 control subjects (boy/girl = 78/74), who were recruited from the same primary school. Mean age was 10.5 ± 1.6 years. Inclusion criteria were: (a) aged 6–14 years; (b) no any ODD symptoms; (c) intelligence quotient (IQ) ≥70 based on Raven’s Progressive Matrices; (d) no physical diseases, mental retardation, low body mass index (BMI, <18.5 kg/m^2^) or other mental illnesses, or ADHD or CD symptoms.

There was no significant differences in gender, age and education between ODD and control groups (all *P* > 0.05). This study was approved by the Ethical Committee of the Second Affiliated Hospital, Xinxiang Medical College, Henan Province. Informed consent was obtained from all subjects and their parents.

### *TPH*-*2* genotyping

5 ml blood samples were collected from cubital vein between 8:00 and 9:00 a. m. following an overnight fast and placed into the tubes with EDTA anticoagulant. Samples were stored at −70 °C until assayed.

DNA was extracted using a Genomic DNA extraction kit (DP318) (TIANGEN biotechnology company, Beijing, China). The DNA was amplified by polymerase chain reaction (PCR) methods and the primers for the four loci (rs4570625, rs11178997, rs1386494 and rs7305115) were designed by Invitrogen Corporation (Shanghai, China). Oligonucleotide sequences are presented in Table 1.

**Table 1:** Primer sequences of these four loci

SNP ID	Primer sequence (5′–3′)
rs4570625	F: 5′-GAACCCTTACCTTTCCTTTG-3′
	R: 5′Acry-TCCACTCTTCCAGTTATTTT-3′
rs11178997	F: 5′-GTGTTCGGGAGCACAATAAT-3′
	R: 5′ Acry -AAGCCTGCCACTGGAAGTT-3′
rs1386494	F: 5′-TGTTTCTCGCAGGTTGTTGG-3′
	R: 5′ Acry-AGCAAATGAATCACAAAGGG-3′
rs7305115	F: 5′-TAGTTGGTTTTTCTGTTGC-3′
	R: 5′Acry-CCCTTTTCTCTTTAGGTGAG-3′

Sequences of the four primers

The total volume of the PCR reaction was 30 μl which contained 0.5 μl whole genome DNA (50 ng/μl), 3 μl 10× PCR buffer solution, 0.5 μl 10 mm L^−1^ dNTPs, 0.5 μl each primer, 0.3 μl *Taq* polymerase, 1.5 μl 25 mm L^−1^ MgCl_2_, and 23.7 μl sterile water. Loop parameters for PCR were as follows: initial denaturation at 95 °C for 5 min, amplification at 94 °C for 30 s, annealing at 54/56 °C for 45 s, and extension at 72 °C for 45 s. The process was repeated 34 times, followed by extension at 72 °C for 5 min. PCR products were placed onto glass slides disposed with acrylamide by a Pixsys5500 microarrayer (Cartesian Products, Inc. America) [33]. The PCR products were hybridized with fluorescence-labeled probes at 37 °C for 5–6 h, and the glass slides were scanned with a LuxScan-10k confocal scanner (Capitalbio Corporation, Beijing, China). The genotype of each sample was detected based on the fluorescent signals [34].

### Statistical analysis

Differences between genotype groups were analyzed using Chi squared for categorical variables and the Student’s t test or one-way analysis of variance (ANOVA) for continuous variables using the PASW Statistics 18.0 software (SPSS Inc., Chicago, IL, USA).

Deviation from the Hardy–Weinberg equilibrium (HWE) was tested separately in cases and controls using Chi square (*χ* ^*2*^) goodness-of-fit test. The difference in the allele and genotype frequencies for *TPH*-*2* polymorphisms between ODD and normal controls was analyzed using the *χ* ^*2*^ test. Pairwise linkage disequilibrium (LD) between four *TPH*-*2* markers was analyzed in cases and normal controls. Haploview 4.2 was used to compute pairwise LD statistics for markers, haplotype block, haplotype frequency, and haplotype association. We used a 2–4-window fashion analysis. Rare haplotypes found in less than 3% were excluded from the association analysis. A logistic regression analysis was conducted to examine the independent association of each haplotype on the categorical diagnosis of case (0: control, 1: case) after adjusting for the confounders. To control haplotype analyses for multiple testing, 10,000 permutations were performed for the most significant tests to determine the empirical significance.

The power (power defined as the chance that true differences will actually be detected) of the sample was calculated using Quanto Software [35], with known risk allele frequencies and an ODD population prevalence of 0.02–0.16, and we examined log additive, recessive and dominant models.

## Results

### Single locus analysis

The genotype and allele frequencies of four SNPs located in the TPH-2 gene are summarized in Table 6. No deviation from HWE was detected in the cases or controls (all *P* > 0.05; Tables 2, 3, 4, 5). Significant differences in the genotype and allele frequencies between cases and controls were observed for rs1386494 (genotype *χ* ^*2*^ = 4.163, *P* = 0.041; allele *χ* ^*2*^ = 3.930, *P* = 0.047). The frequency of the Gallele of rs1386494 was higher in patients than in controls. There was no allelic or genotypic association between the other three SNPs and ODD (all *P* > 0.05, Table 6).

**Table 2:** rs4570625 Hardy–Weinberg Equilibrium test between ODD and control group

Group	Genotype					
	GG	GT	TT	Total	χ^2^	*P*
ODD group					1.474	0.225
Observation (O)	27	51	38	116		
Expectation (E)	23.761	57.478	34.761	116		
Control group					1.301	0.254
Observation (O)	36	83	33	152		
Expectation (E)	39.515	75.970	36.51	152		

df = 1

**Table 3:** rs11178997 Hardy–Weinberg equilibrium test between ODD and control group

Group	Genotype						
	AA	AT	TT	Total	χ^2^	χ^2*^	*P*
ODD group						1.292	0.256
Observation (O)	6	33	84	123			
Expectation (E)	4.116*	36.768	82.116	123			
Control group						0.052	0.820
Observation (O)	4	39	109	152			
Expectation (E)	3.633*	39.734	108.63	152			

df = 1, when E is little than 5, we use Yates corrected Chi squared test, χ^2*^

**Table 4:** rs1386494 Hardy–Weinberg equilibrium test between ODD and control group

Group	Genotype						
	AA	AG	GG	Total	χ^2^	χ^2*^	*P*
ODD group						0.138	0.711
Observation (O)	0	8	116	124			
Expectation (E)	0.129*	7.742	116.129	124			
Control group						0.856	0.355
Observation (O)	0	21	128	149			
Expectation (E)	0.740*	19.520	128.740	149			

df = 1* when E is little than 5, we use Yates corrected Chi squared test, χ^2*^

**Table 5:** rs7305115 Hardy–Weinberg equilibrium test between ODD and control group

Group	AA	AG	GG	Total	χ^2^	*P*
ODD group					3.389	0.533
Observation (O)	34	56	29	119		
Expectation (E)	32.303	59.395	27.302	119		
Control group					0.844	0.358
Observation (O)	35	64	40	139		
Expectation (E)	2.295	69.410	37.295	139		

df = 1

**Table 6:** Genotype and allele frequency distribution of the four loci of *THP*-*2* between two groups

Locus	Genotype (%)			*χ* ^*2*^	*P*	Allele (%)		*χ* ^*2*^	*P*
rs4570625	GG	GT	TT	4.525	0.104	G	T	1.729	0.189
ODD	27 (23.3)	51 (44.0)	38 (32.8)			105 (45.3)	127 (54.7)		
Control	36 (23.7)	83 (54.6)	33 (21.7)			155 (51.0)	149 (49.0)		
rs11178997	AA	AT	TT	1.092	0.579	A	T	0.783	0.376
ODD	6 (4.9)	33 (26.8)	84 (68.3)			45 (18.3)	201 (81.7)		
Control	4 (2.6)	39 (25.7)	109 (71.7)			47 (15.5)	257 (84.5)		
rs1386494	AA	AG	GG	4.163	0.041*	A	G	3.930	0.047*
ODD	0	8 (6.5)	116 (93.5)			8 (3.2)	240 (96.8)		
Control	0	21 (14.1)	128 (85.9)			21 (7.0)	277 (93.0)		
rs7305115	AA	AG	GG	0.756	0.685	A	G	0.780	0.377
ODD	34 (28.6)	56 (47.1)	29 (24.4)			124 (52.1)	114 (47.9)		
Control	35 (25.2)	64 (46.0)	40 (28.8)			134 (48.2)	144 (51.8)		

* P<0.05

### Linkage disequilibrium (LD) analysis

**Fig. 1:**
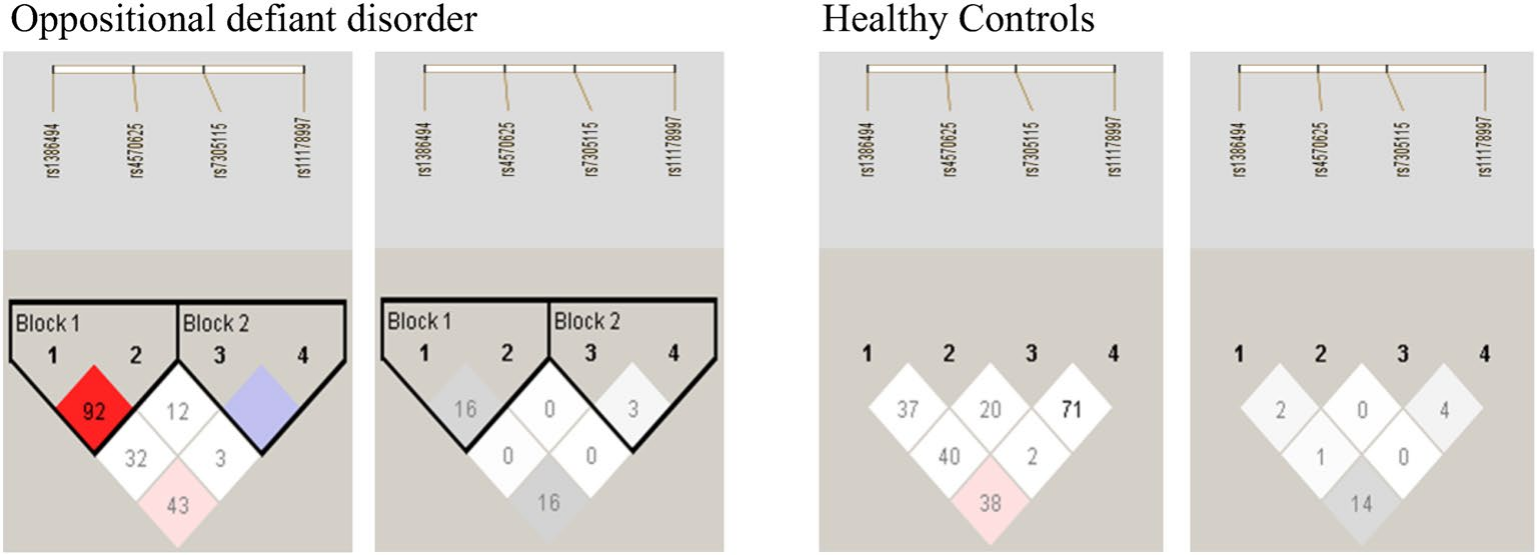
Genomic structure of *TPH*-*2*, including relative location of 4 SNPs studied and linkage disequilibrium (LD) of these four SNPs in the oppositional defiant disorder (ODD) and control groups. The LD between pairwise SNPs, using D′ (*left*, *red color*) and r^2^ (*right*, *gray color*) values, are shown separately for cases and controls. High levels of LD are represented by increasing scale intensity from 0 to 100, as shown by the *bars*

LD analyses were performed for all polymorphism pairs in both case and control subjects. All four polymorphisms were in slight to modest LD or without LD with each other in both control (D′ = 0.12–0.92; r^2^ = 0.02–0.71) and patient groups (D′ = 0.10–0.76; r^2^ = 0.00–0.16) (Fig. 1).

### Haplotype analysis

Two–four SNP sliding window haplotype analyses were performed. Only those haplotypes with a frequency above 3% were included in the analyses. Estimation of haplotype frequencies and comparison of haplotype frequency distributions between cases and controls were conducted using the program Haploview.

We observed significant differences in the frequencies of TA (*P* = 0.014, *OR* = 1.951, 95% *CI* 1.140–3.341) and GA (*P* = 0.012, *OR* = 0.149, 95% *CI* 0.027–0.826) haplotypes containing rs4570625-rs11178997 between case and control groups.

Also, we noted significant differences in the frequencies of TAG (*P* = 0.02, *OR* = 1.896, 95% *CI* 1.099–3.272) and GAG (*P* = 0.013, *OR* = 0.149, 95% *CI* 0.027–0.831) containing rs4570625-rs11178997-rs1386494, as well as TAA (*P* = 0.026, *OR* = 2.315, 95% *CI* 1.088–4.927) containing rs4570625-rs11178997-rs7305115 between case and control groups.

Finally, we found significant differences in the frequencies of TAGA (*P* = 0.005, *OR* = 3.187, 95% *CI* 1.376–7.382) containing rs4570625-rs11178997-rs1386494-rs7305115 between ODD and control groups (Tables 7, 8).

**Table 7:** Analysis of genetic linkage disequilibrium of the four loci

SNPs	SNP2		SNP3		SNP4	
	D′	r2	D′	r2	D′	r2
SNP1	0.702	0.090	0.093	0.001	0.405	0.161
SNP2			0.653	0.005	0.001	0.000
SNP3					0.887	0.049

SNP1:rs4570625; SNP2:rs11178997; SNP3:rs1386494; SNP4:rs7305115

**Table 8:** Haplotype analysis of *TPH*-*2* between two groups

Haplotype distribution	Case (freq)	Control (freq)	*χ* ^*2*^	Fisher *P*	OR [95% CI]
SNP1-2					
G-A	1.52 (0.007)	10.57 (0.042)	6.281	0.012233*	0.149 [0.027–0.826]
T-A	40.48 (0.174)	24.43 (0.098)	6.083	0.013679*	1.951 [1.140–3.341]
SNP1-2-3					
G-A-G	1.51 (0.007)	10.51 (0.042)	6.234	0.012562*	0.149 [0.027–0.831]
T-A-G	38.73 (0.167)	24.00 (0.096)	5.409	0.020075*	1.896 [1.099–3.272]
SNP1-2-4					
T-A-A	22.24 (0.096)	10.60 (0.042)	4.981	0.025669*	2.315 [1.088–4.927]
SNP1-2-3-4					
T-A-G-A	21.77 (0.094)	7.79 (0.031)	8.031	0.004617*	3.187 [1.376–7.382]

SNP1:rs4570625; SNP2:rs11178997; SNP3:rs1386494; SNP4:rs7305115 * P<0.05

However, the further analysis by Haploview revealed the nominally significant finding for rs1386494 (*χ* ^*2*^ = 3.846, *P* = 0.0499), and only one haplotypes (TAGA, *χ* ^*2*^ = 4.366, *P* = 0.0367) remained significant. However, the results did not remain statistically significant after 5000-fold permutation-based analysis incorporating all four SNPs or all the observed haplotypes (adjusted both *P* > 0.14). Thus, our study was only considered as preliminary evidence of a possible association.

### Power analysis

This total sample had 0.10–0.47 power, 0.31–0.70 power, and 0.36–0.97 power for these four polymorphisms to detect recessive, log additive and dominant polymorphic inheritance in ODD with an odds ratio (OR) of 2 or greater (alpha = 0.05, two tailed test).

## Discussion

To our knowledge, this is the first study to find an association between ODD and the TPH-2 gene polymorphism rs1386494 or the haplotype formed by this polymorphism and other polymorphisms. While most *TPH*-*2* association studies have used individual markers, we used polymorphism-based haplotypes and LD analysis to show that both ODD and controls shared a homogeneous LD pattern. This LD suggests that these variants segregate together in a Chinese population.

There is an extensive data consistently showing that decreased functions of the central 5-HT activity are associated with uncontrolled behaviors, including impulsive behavior, aggressiveness, and substance abuse both in humans and in animal models, for example [29, 36, 37]. TPH-2, a rate-limiting enzyme in the biosynthesis of 5-HT, is expressed mainly in brain [38]; thus, it influences the 5-HT level in brain and plays an important role in the development of mental disease [16]. Genetic variation in *TPH*-*2* activity is likely to represent a critical factor in the pathogenesis of ADHD and impulsivity [16, 23]. Several studies have demonstrated that changes in the 5-HT system were critical in children with ODD [39, 40]. Moreover, the polymorphisms of *TPH*-*2* have been shown to be associated with ADHD [16, 41, 42], obsessive–compulsive disorder [43], and bipolar affective disorder [44]. Also, *TPH*-2 was found to be associated with major depression [45] and pathogenesis of depression in Chinese females [46] and suicidal behavior [47, 48]. However, there has been no study reporting the relationship between *TPH*-*2* and ODD.

This study is a case–controlled study in which the frequency of the genotypes and alleles of *TPH*-*2* polymorphisms were compared between the ODD children and the control group in Chinese Han. The association between the genotypes and alleles of four candidates *TPH*-*2* SNPs was investigated. Our results showed that there was a significant correlation between the frequencies of the *TPH*-*2*-rs1386494 and ODD, but not other three polymorphisms, suggesting that the locus of rs1386494 of *TPH*-*2* was associated with ODD in Chinese Han children. The association of *TPH*-*2*-rs1386494 in this study was inconsistent with the result of Walitza et al. [23] study, in which loci of rs4570625 and rs11178997 were found to be associated with ADHD and combined ADHD and ODD, but not rs4565946. There are several reasons to explain the inconsistent results. First, the subjects in Walitza’s study included both ADHD and ODD. Second, the linkage disequilibrium analysis in the core family was used in Walitza’s study while the case-control association was used in several studies including our current study. Thus, the differential analysis methods and different samples may contribute to discrepant results.

Haplotypes can be more specific risk markers than single alleles, and their use reduces false-positive associations that can occur because common psychiatric disorders are likely to associate with common alleles [49]. Since the four markers analyzed were in the same haplotype block, we performed the two–four SNP sliding window haplotype analysis [50]. We found that the frequencies of the TA and GA haplotypes containing rs4570625-rs11178997, the TAG and GAG haplotypes containing rs4570625-rs11178997-rs1386494, the TAA haplotype containing rs4570625-rs11178997-rs7305115, and the TAGA haplotype containing rs4570625-rs11178997-rs1386494-rs7305115 were significantly different between ODD and controls (all *P* < 0.05). Further, TA, TAG, TAA and TAGA haplotypes might be the risk factors for ODD, while GA and GAG haplotypes might be protective for ODD in Chinese Han children.

Several limitations of this study should be noted. First, the number of subject children was small. The subjects of this study were 124 ODD children and 152 children in the control group. Since our sample size provided only poor statistical power, it is possible that we do see the false-positive results in the present study and our findings need to be considered cautiously. A replication study would be needed to include a large sample size. Second, although we had genotyped four polymorphisms in the present study, the coverage of genetic variation is too limited considering the total *TPH*-*2* gene variants includes at least 50 polymorphisms. Therefore, it would be much better to use GWAS in larger samples to capture true positive results found in our present study. Third, the samples in our current study were only from local city in Henan province, China. Thus, the findings of this study may not be generalized for the cases of other racial or ethnic groups since the frequency of alleles can vary due to local or racial differences. Fourth, it would be better to use DSM-V as a reference rather than DSM-IV. Unfortunately, we had no DSM-V when our current study was conducted. We will use DSM-V as a reference in our future investigation to remedy this shortcoming.

In summary, we report several convergent findings that implicate an effect of *TPH*-*2* genotype on increased risk for ODD. We found a potential genetic association of *TPH*-*2* with risk for ODD, especially the *TPH*-*2* gene polymorphism rs1386494. Further haplotype analyses showed that TA, TAG, TAA and TAGA haplotypes might be the risk factors for ODD, while GA and GAG haplotypes might be protective for ODD in Chinese Han children. However, the findings in our present study remain preliminary due to the limited sample size and our low statistical power, as well as poor coverage of genetic variations in *THP*-*2*, which require replication in larger samples of ODD children from different ethnic populations.

## Appendix

**Table:** ODD group

Sex (1=boy;2=girl)	Age	rs4570625	rs11178997	rs1386494	rs7305115
1	8	GT	AT	GG	AG
2	6	TT	AT	GG	AG
1	7	GT	AT	GG	AG
1	8	TT	AT	GG	AA
2	9	GG	TT	GG	GG
1	9	GT	TT	GG	AA
2	7	TT	TT	GG	AA
1	10	TT	TT	AG	AA
1	13	TT	AT	GG	AG
1	10	GT	TT	GG	AG
2	10	GT	TT	GG	AG
2	9	TT	AT	GG	AA
2	7	GT	TT	GG	AG
2	9	GG	TT	GG	GG
2	10	GT	TT	AG	AG
2	10	TT	TT	GG	AA
1	9	GT	TT	GG	AG
2	10	GT	TT	GG	GG
1	8	GG	TT	GG	GG
2	9	GG	TT	GG	GG
1	10	GG	TT	GG	GG
2	12	TT	AA	GG	AG
1	10	TT	AT	GG	AA
1	9	GG	TT	GG	AG
2	10	GT	AT	GG	GG
2	10	GT	AT	AG	AA
2	9	TT	AT	GG	AA
1	11	TT	TT	GG	AA
1	14	TT	AT	GG	AG
2	14	TT	AA	AG	AG
1	12	TT	TT	GG	AA
2	14	GT	TT	GG	AG
2	9	GT	AT	GG	AG
1	10	GT	TT	GG	AG
1	11	GG	TT	GG	AG
1	12	GG	TT	AG	AG
1	10	TT	TT	GG	AG
2	12	GT	TT	GG	AG
2	12	GG	TT	GG	AG
2	13	GT	AT	GG	AG
1	11	GT	AT	AG	AG
2	8	GT	AT	GG	GG
2	8	GT	TT	GG	AG
1	8	GT	TT	GG	AG
2	12	GG	TT	GG	GG
1	9	GG	TT	AG	AA
1	11	GT	TT	GG	AG
1	11	GT	TT	GG	AA
1	9	TT	AA	GG	AG
2	10	TT	TT	GG	AA
1	7	TT	TT	GG	AA
2	7	GT	TT	GG	GG
1	11	GT	AT	GG	GG
1	10	GT	TT	GG	AG
2	11	GT	TT	GG	AG
1	10	TT	TT	GG	GG
1	11	TT	TT	GG	AA
2	12	GT	TT	GG	AA
2	10	GG	AT	GG	GG
1	12	GT	AT	GG	AG
1	14	TT	TT	GG	GG
1	7	GG	TT	AG	AA
2	8	TT	AA	GG	AA
2	9	GT	TT	GG	AA
2	12	TT	TT	GG	AA
1	9	TT	AT	GG	GG
1	9	GG	TT	GG	AG
2	9	GG	TT	GG	AG
2	12	TT	AT	GG	GG
2	11	TT	TT	GG	AG
1	10	GG	TT	GG	GG
2	11	GG	TT	GG	GG
2	10	GG	TT	GG	AG
1	12	GT	TT	GG	AG
2	11	GT	TT	GG	AG
2	11	GT	TT	GG	AG
1	9	GG	TT	GG	GG
2	8	GT	TT	GG	AG
1	12	GT	AT	GG	AA
1	12	GT	TT	GG	AA
2	7	GT	AT	GG	AG
1	14	TT	TT	GG	AA
1	12	GT	TT	GG	AA
1	10	GT	TT	GG	AG
1	12	TT	TT	GG	AA
2	12	GG	TT	GG	AG
1	11	TT	AT	GG	AA
1	13	TT	TT	GG	AG
1	14	GT	TT	GG	AG
2	8	GG	TT	GG	AG
1	8	GT	TT	GG	AG
2	13	GT	AT	GG	AG
2	10	GG	TT	GG	GG
1	13	GG	TT	GG	GG
2	8	GG	TT	GG	GG
2	9	TT	AA	GG	GG
1	8	GT	TT	GG	AG
2	13	TT	TT	GG	AA
1	13	GT	AT	GG	AG
1	9	TT	AT	GG	GG
2	11	GT	TT	GG	GG
1	13	GG	TT	GG	GG
2	10	GT	TT	GG	AA
1	10	TT	AA	GG	AG
2	10	GT	TT	GG	AG
1	8	GG	TT	GG	AG
1	9	GT	TT	GG	AA
1	9	TT	AT	GG	AA
1	12	GG	TT	GG	GG
2	13	TT	TT	GG	AG
1	9	TT	AT	GG	AG
1	10	GT	TT	GG	AA
1	12	GT	AT	GG	GG
1	13	GT	AT	GG	AA
1	12	GT	AT	GG	GG
1	13	TT	TT	GG	AG
1	11		AT	GG	AA
1	12		AT	GG	AG
1	11		TT	GG	AG
2	12		TT	GG	
1	12		TT	GG	
1	11		TT	GG	
1	12		TT	GG	
1	13			GG

**Table:** Control group

Sex (1=boy;2=girl)	Age	rs4570625	rs11178997	rs1386494	rs7305115
1	8	GG	TT	GG	GG
2	7	TT	TT	GG	AA
1	7	GT	TT	AG	AA
2	9	GT	TT	GG	AG
2	10	GT	TT	GG	AG
1	9	TT	TT	GG	AA
2	8	GT	TT	GG	AG
1	8	GT	TT	AG	AG
1	11	GG	TT	GG	GG
1	9	GG	TT	GG	GG
2	10	TT	AT	AG	AA
2	10	GG	TT	GG	GG
2	13	GT	TT	GG	GG
1	11	TT	TT	GG	AA
1	13	TT	TT	GG	GG
2	7	GT	TT	GG	AG
1	12	GT	TT	GG	AG
1	14	GT	AT	GG	AA
1	10	GG	TT	AG	AA
1	12	GT	TT	GG	GG
2	12	TT	AT	GG	AG
2	11	GG	TT	AG	AG
2	11	GG	TT	GG	GG
2	9	GG	TT	GG	AA
2	11	GT	TT	GG	AG
2	12	GT	AT	GG	AA
2	10	GT	TT	GG	AA
1	13	GT	TT	GG	AA
1	9	GG	TT	GG	GG
1	9	GT	TT	GG	AG
1	8	GT	TT	GG	AA
2	7	TT	AT	GG	AG
2	10	TT	TT	GG	AA
1	11	GT	TT	AG	AG
1	12	GT	TT	GG	AA
2	10	GG	TT	GG	GG
1	12	TT	AT	GG	AG
2	11	TT	TT	GG	GG
2	11	GG	TT	GG	AG
2	11	GG	TT	GG	AG
1	11	GG	TT	GG	GG
2	12	TT	TT	GG	GG
2	10	GT	AT	GG	AA
1	9	GG	TT	AG	AG
1	11	GT	TT	GG	AG
2	12	GG	AA	GG	AG
1	12	GT	AT	GG	GG
2	12	GT	TT	GG	AG
2	12	GT	TT	GG	AG
2	9	GT	TT	GG	GG
1	9	GT	TT	GG	AG
1	11	TT	AA	GG	AG
2	14	GT	TT	GG	AG
1	12	GG	TT	GG	AG
1	12	GT	AT	GG	AG
1	12	TT	TT	GG	AA
1	11	GG	TT	GG	AG
1	12	GT	TT	GG	AG
1	12	GG	AT	GG	AG
1	12	GT	AT	GG	GG
1	12	TT	TT	GG	AG
1	12	TT	TT	GG	AA
1	12	GG	AT	GG	AG
1	11	GT	AT	GG	AG
1	10	GG	TT	GG	GG
2	11	GT	AT	GG	GG
2	13	GT	TT	GG	AA
2	13	GT	TT	GG	AG
2	12	TT	AT	GG	AG
2	13	TT	TT	GG	GG
2	12	GG	TT	GG	GG
2	11	GT	TT	AG	AA
2	10	GG	TT	GG	GG
1	10	GT	TT	GG	AA
2	10	TT	AT	AG	AA
2	11	GT	TT	AG	AA
2	10	GT	AT	GG	AG
2	10	GT	AT	GG	AG
2	9	GT	TT	GG	AG
2	10	GG	TT	GG	GG
2	9	TT	AT	GG	AA
2	10	GT	AT	GG	GG
2	10	TT	TT	GG	AA
1	8	GT	TT	GG	AG
1	9	TT	TT	AG	AA
2	10	TT	TT	GG	GG
2	9	GT	TT	GG	AG
1	10	GT	AT	AG	AG
1	11	TT	TT	AG	AG
1	12	GT	TT	GG	AG
2	13	GT	TT	GG	AA
2	10	GG	TT	GG	AG
2	11	GT	AT	GG	GG
1	11	GT	TT	AG	AG
2	11	TT	TT	GG	AA
1	11	GT	TT	GG	AG
2	13	GG	TT	GG	AG
1	13	TT	TT	GG	AG
2	13	GT	TT	GG	AG
1	11	TT	AT	GG	AG
2	10	GG	TT	GG	GG
2	8	GT	TT	GG	AG
2	9	GT	TT	GG	AG
1	9	GT	AT	GG	GG
1	10	GT	AT	GG	GG
1	11	GT	TT	GG	AG
1	10	GT	AT	GG	AG
1	11	GT	AT	GG	AG
2	10	GG	TT	GG	GG
1	9	GT	TT	AG	AA
2	12	GT	AA	GG	AG
2	12	GT	TT	GG	AG
1	11	GT	TT	GG	AG
1	7	GT	TT	GG	AG
1	10	TT	TT	GG	AA
1	8	GG	TT	GG	GG
1	11	GT	TT	GG	AG
2	13	GG	TT	GG	GG
1	12	GG	AT	GG	AA
1	10	GG	TT	GG	AA
1	10	GT	TT	GG	GG
2	8	GT	TT	GG	AG
2	8	GT	AT	AG	AG
1	12	GG	TT	GG	GG
1	12	GG	TT	GG	GG
2	7	TT	TT	GG	AG
1	7	GT	TT	AG	GG
2	9	GT	AT	GG	GG
2	10	GT	AT	AG	AG
1	9	TT	TT	GG	AA
2	10	GT	AT	GG	GG
1	8	GT	AT	AG	GG
1	11	GG	TT	GG	GG
1	9	GG	AT	GG	AA
2	10	TT	TT	AG	AA
2	10	GT	TT	GG	GG
2	13	GT	TT	GG	AG
1	11	TT	AT	GG	AG
1	11	TT	TT	AG	AA
2	7	GT	TT	GG	
1	12	GT	TT	GG	
1	11	GT	AT	GG	
1	10	GT	TT	GG	
1	12	GT	AT	GG	
1	12	TT	AT	GG	
2	11	GT	AT	GG	
1	11	GT	AT	GG	
2	9	GG	TT	GG	
1	11	GT	TT	GG	
2	12	GT	TT		
2	10	GT	TT		
1	8	GT	AA		

## Abbreviations

ODD: oppositional defiant disorder
CD: conduct disorder
ADHD: attention-deficit/hyperactivity disorder
*TPH*-*2*: the tryptophan hydroxylase-2 gene
5-HT: serotonin
IQ: intelligence quotient
PCR: polymerase chain reaction
SNP: single nucleotide polymorphisms
